# Integration of Full-Size Graywater Membrane-Aerated Biological Reactor with Reverse Osmosis System for Space-Based Wastewater Treatment

**DOI:** 10.3390/membranes14060127

**Published:** 2024-05-30

**Authors:** Ghaem Hooshyari, Arpita Bose, W. Andrew Jackson

**Affiliations:** Department of Civil, Environmental, and Construction Engineering, Texas Tech University, 911 Boston Avenue, Lubbock, TX 79409, USA; ghaem.hooshyari@ttu.edu (G.H.);

**Keywords:** biological pretreatment, graywater, Reverse Osmosis, membrane-aerated biological reactor (MABR), space habitation wastewater, life support system, water recovery

## Abstract

To date, life support systems on the International Space Center (ISS) or those planned for upcoming moon/Mars missions have not included biological reactors for wastewater treatment, despite their ubiquitous use for the treatment of terrestrial wastewaters. However, the new focus on partial gravity habitats reduces the required complexity of treatment systems compared with those operating in micro-gravity, and the likely addition of large-volume wastewaters with surfactant loads (e.g., laundry and shower) makes the current ISS wastewater treatment system inappropriate due to the foaming potential from surfactants, increased consumable requirements due to the use of non-regenerative systems (e.g., mixed adsorbent beds), the complexity of the system, and sensitivity to failures from precipitation and/or biological fouling. Hybrid systems that combine simple biological reactors with desalination (e.g., Reverse Osmosis (RO)) could reduce system and consumable mass and complexity. Our objective was to evaluate a system composed of a membrane-aerated bioreactor (MABR) coupled to a low-pressure commercial RO system to process partial gravity habitat wastewater. The MABR was able to serve as the only wastewater collection tank (variable volume), receiving all wastewaters as they were produced. The MABR treated more than 20,750 L of graywater and was able to remove more than 90% of dissolved organic carbon (DOC), producing an effluent with DOC < 14 mg/L and BOD < 12 mg/L and oxidizing >90% of the ammoniacal nitrogen into NOx_−_. A single RO membrane (260 g) was able to process >3000 L of MABR effluent and produced a RO permeate with DOC < 5 mg/L, TN < 2 mg/L, and TDS < 10 mg/L, which would essentially meet ISS potable water standards after disinfection. The system has an un-optimized mass and volume of 128.5 kg. Consumables include oxygen (~4 g/crew-day), RO membranes, and a prefilter (1.7 g/crew-day). For a one-year mission with four crew, the total system + consumable mass are ~141 kg, which would produce ~15,150 kg of treated water, resulting in a pay-back period of 13.4 days (3.35 days for a crew of four). Given that the MABR in this study operated for 500 days, while in previous studies, similar systems operated for more than 3 years, the total system costs would be exceedingly low. These results highlight the potential application of hybrid treatment systems for space habitats, which may also have a direct application to terrestrial applications where source-separated systems are employed.

## 1. Introduction

The International Space Station (ISS) has operated for 24 years and currently supports up to seven inhabitants [1]. NASA is also currently developing a moon habitat to allow astronauts to live and work on the moon’s surface for extended periods. This moon habitat is seen as a steppingstone to an eventual Mars habitat [2]. One of the most significant challenges for human space travel and habitation is the life support requirements, particularly water availability. Short-term missions like the Space Shuttle, Apollo, and the planned space launch system (Orion Artemis) used stored water and oxygen [3]. However, resupplying water from Earth for longer-term missions is not feasible due to both costs and the inability to reliably ship water to distant destinations [4]. Resupply is not considered a viable option even for the ISS in low Earth orbit (LEO), and the ISS extensively utilizes water recycling [5]. Recycling water is necessary for extended missions such as the Mars mission [6,7]. The need for potable water during space travel, coupled with the inability to easily resupply during missions away from LEO [8] and the cost of resupply, has driven the development of wastewater treatment technologies that produce potable water using minimal consumables [9].

On the ISS, urine + flush water (U+F) (~1.8 L and ~0.3 L /crew-day) and humidity condensate (HC) (~2 L/crew-day) are treated to provide potable water with an overall recovery rate of ~ 85% [10]. The U+F water is pretreated using phosphoric acid (H_3_PO_4_) and chromic acid (H_2_CrO_4_) in order to prevent urea hydrolysis, which would cause the pH to increase and produce NH_3_ (which would be lost to the cabin) and also cause calcium sulfate (CaSO_4_) precipitation [11]. The U+F is treated using a microgravity-compatible distillation system (urine process assembly (UPA)), and solids production has caused failures [12,13]. After distillation, the distillate and HC are combined in a water process assembly (WPA) system storage tank [14]. The waste stream is initially degassed and pumped through a 0.5-micron filter and Multifiltration Bed (MFB), which contains ion exchange and adsorbent media. Biological growth in the WPA storage tank has caused failures due to the growth of biomass. Volatile organics not effectively removed by the MFB are oxidized in the catalytic reactor at elevated temperatures, which also removes microbial contamination [14]. Dissolved byproducts are removed by the ion exchange bed [10].

To date, the ISS water recycling system has been very successful. Approximately 7500 L of product water has been produced yearly since the system was implemented [9,10]. The ISS’s WPA is gravity-independent but requires substantial consumables (e.g., MFB and pretreat chemicals) and produces a brine that creates additional challenges regarding the storage, dewatering, and disposal of residual solids [15]. In addition, for partial gravity habitat (PGH) waste streams, the ISS treatment system cannot utilize the benefits of gravity and is not compatible with future long-term mission wastewaters, such as hygiene (tooth-washing waste and shaving), shower, and laundry, some of which are large in volume in comparison with ISS wastewaters and have the potential to produce foam due to the presence of surfactants.

For future space travel focused on partial gravity habitations (PGHs) (e.g., moon or Mars), with no or limited resupply, consumable mass must be minimized, and sustainable human habitation will require independence from resupply [10,16]. The PGH waste stream includes U+F; humidity condensate; and potentially shower, hygiene activities, and laundry [17]. Partial gravity enables the use of additional technologies and configurations that can be inherently more robust as there is no requirement for one-phase flow; gravity-dependent technologies (e.g., sedimentation) are possible, and systems can be engineered to allow precipitates. Therefore, other methods of wastewater processing may reduce the need for hazardous chemicals and consumables and allow for the inclusion of other waste streams.

Biological treatment is an alternative low-cost and reliable process that can eliminate the need for hazardous chemicals and treat all proposed wastewaters [17,18]. Biological systems have been shown to maintain an appropriate pH (5–7) for desalination systems, provide a stable effluent that minimizes further microbial growth by removing organic carbon, and convert organic N into nitrogen gas (N_2_) or NO_x_^−^ [17,19]. Membrane-aerated biological reactors (MABRs) can continuously treat habitation wastewaters (e.g., HC, U+F, hygiene, shower, and laundry waste) for up to 5 years, removing 90% of organic carbon and converting up to 70% of organic N [18,19]. However, these systems have yet to be evaluated for treating graywater. Separating graywater from U+F could produce a high-quality, high-volume resource amenable to RO while a distillation system desalinates the low-quality, low-volume U+F. However, to the best of our knowledge, studies have yet to evaluate hybrid treatment systems for habitation wastewater.

**Figure 1:**
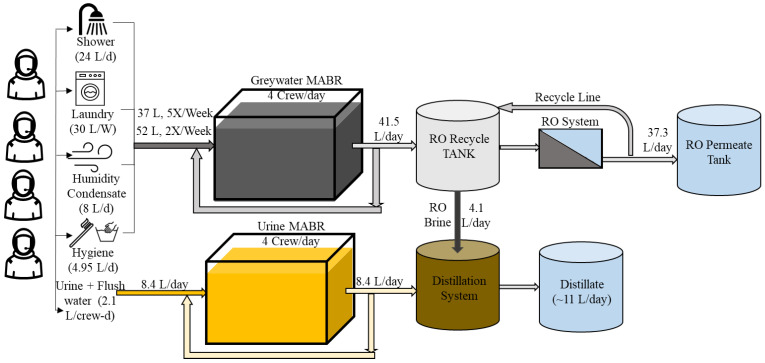
Simplified process flow diagram for proposed hybrid habitation wastewater treatment system. Flow rates reflect a load of four crew members per day. The graywater MABR receives 37 L of graywater, excluding laundry five times per week and 52 L two times per week, which includes laundry loads in addition to graywater. The graywater effluent (~41.5 L) discharges as a pulse to the RO system recycle tank once daily. The urine reactor receives 8.4 L of urine + flush water over an 18 h period (wake cycle) and an estimated 24 urine events. The brine from RO processing and urine reactor effluent are transferred to the distillation system for treatment.

Hybrid wastewater processing systems that include both biological pretreatment and desalination provide multiple benefits that address these challenges. Figure 1 displays one potential hybrid treatment train for recycling graywater and U+F. It consists of variable-volume, full-scale bioreactors; RO; and a distillation system. In this system, there are two biological reactors to separately treat the U+F and all other graywaters (e.g., hand washing, shaving, showering, toothpaste, and laundry waste). The bioreactors serve as both collection tanks and treatment systems in which the waste is stabilized by converting the organic carbon into CO_2_ and organic N into NO_x_^−^ (NO_3_^−^ + NO_2_^−^), which also lowers the pH and reduces the loss of NH_3_ due to volatilization. Using bioreactors as collection tanks also eliminates the need for separate collection vessels. The RO system treats effluents from the graywater bioreactor. The brine produced by the RO system is transferred into a static distillation system, which also processes the effluent from the MABR treating the U+F. For this configuration, the bioreactors are membrane-aerated bioreactors that use dense non-porous membranes to provide O_2_ by diffusion directly into the biofilm and bulk liquid. These systems have been extensively evaluated for space-based wastewaters and have numerous advantages, such as reduction in foaming, minimal evaporation and volatile loss (e.g., odor or NH_3_), very low solids production, and the ability to use pure O_2_ [19,20].

**Figure 2:**
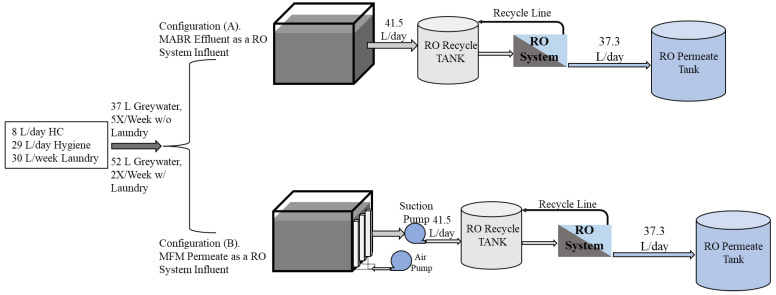
Flow diagram of each process configuration for the graywater–RO system.

This study evaluated a full-size hybrid system (Figure 1) processing a habitation waste stream. This paper only focuses on the graywater MABR-RO system’s results. System performance in terms of processing capacity, rate, consumables, and water quality was evaluated for two configurations (Figure 2A,B). The MABR was directly fed with wastewaters as they were produced for all systems. In system A, the MABR effluent was released once per day to an RO system that operated in batch mode, with the final brine transferred to the distillation system. In system B, a membrane filtration module (MFM) was included within the graywater MABR effluent zone, and the produced permeate was transferred to the RO system once per day for processing as above. Here, we report on the performance of each configuration and the required mass, volume, and consumable requirements. This work provides critical data to allow hybrid systems to be evaluated for partial gravity habitation systems and potentially increase the sustainability of human space habitation.

## 2. Materials and Methods

### 2.1. Graywater MABR and Reverse Osmosis (RO) System Configuration and Operation

The integrated treatment unit comprises a graywater MABR and an RO system (Figure 1). Figure 2 shows the two different graywater MABR and RO system configurations evaluated. In the first configuration (A), the graywater MABR effluent was transferred to the RO system as a pulse once daily (from day 148 to 380). The RO system operated in batch mode until 90% recovery, and the brine (10% of the influent) was drained to the distillation vessel. For the second test point (B), three hollow fiber microporous membrane filtration modules (MFMs) were installed in the MABR effluent zone to achieve better effluent quality. Graywater MABR effluent was produced continuously and collected in the RO system recycle tank (from day 385 to 490).

#### 2.1.1. The Graywater Membrane Aerated Biological Reactor Characteristics

**Figure 3:**
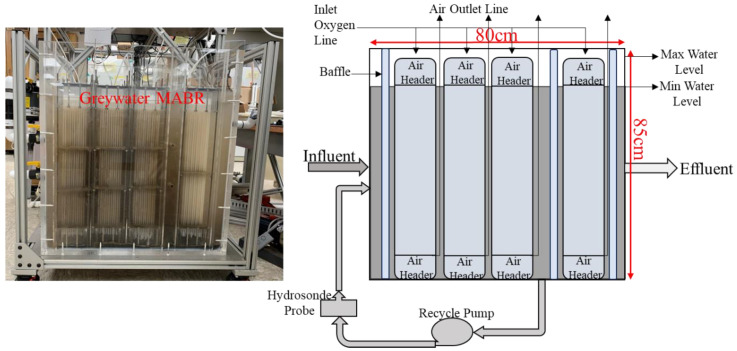
Graywater MABR schematic (80 × 85 × 45 cm), flow diagram, and picture.

Based on previous studies, the graywater MABR was designed and built to have sufficient capacity for four crew members, and the required nominal retention time was 4 days on average. Figure 3 shows the graywater MABR details and characteristics. The loading of the reactor occurred as the wastewater was produced, reflecting how the system would operate if it was the only storage tank. Based on this, the reactor’s total and minimum wet volumes were designed as 238 and 169 L, respectively. The reactor comprises four independent siloxane membrane aeration modules with an inlet and outlet air header per module. Each module contained 374 hollow non-porous siloxane tubes with an ID and OD of 0.245 cm and 0.55 cm, respectively. All four headers received oxygen at 100 mL/min, regulated by mass flow controllers. The specific surface area of the MABR was ~100 m^2^/m^3^. A recycle pump (Goulds Water Technology, Auburn, NY, USA) operated at 22 L/min provided mixing in the reactor. The effluent was manually discharged from the effluent zone of the reactor once per day by gravity.

#### 2.1.2. Reverse Osmosis (RO) System Characteristics

**Figure 4:**
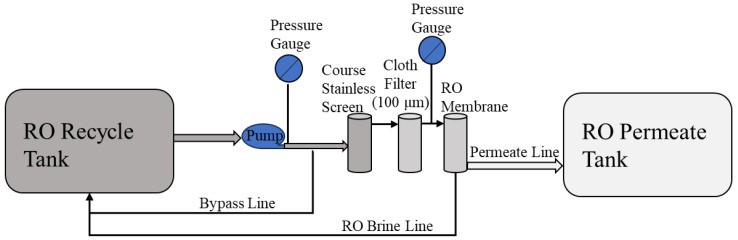
Reverses Osmosis system flow diagram.

The RO system (Figure 4) comprises a 115 L conical recycle tank, a ClearPath recycle pump (MAP V2), a course stainless screen, a cloth prefilter module (Polypropylene fiber, 100 μm), an RO unit, and a 115 L conical permeate tank. Two different commercial RO membrane modules (DOW (FilmTec BW60-1812-75) and Aquaporin Inside^®^ (DWRO 1812)) were evaluated. These two membranes are referred to as DOW and Aquaporin for the rest of this study. Both membranes are of spiral wound construction with a patented Aquaporin coating intended to provide improved rejection of lightweight organics.

#### 2.1.3. Graywater Stream Composition and Feeding Regime

The graywater stream was prepared based on expected volumes and compositions in consultation with NASA. The graywater consists of HC, shower, hygiene, and laundry waste. Table 1 summarizes the daily volume per crew member and constituents of each waste stream. Current estimates state that, for a habitation system serving a crew of four, laundry would be washed two times per week using 15 L of water per event and 34 g of detergent. For this testing, we used Tide Infinity, a detergent specially formulated for extremely low-volume clothes washing and biological treatment of laundry effluent [21]. To produce laundry effluent, lab workers brought personal clothes (4.5 kg/run), which were washed using a programmable LG washing machine with a high RPMS spin cycle on a low flow setting. A prior NASA ersatz HC recipe was initially used to prepare HC [19], but based on more current ISS data [22,23], the procedure was changed to a new formulation (Table S1). The shower wastewater (6 L/crew-day) was produced by washing 2.5 kg of dirty clothes to mimic shower waste and using NO-Rinse shampoo (Sulfochem B-NBBSB-ISS Folmula) (7.5 g of shampoo to 35 L of RO water). Other components included 1 L/crew-day of hand wash (0.21 g of NO-Rinse shampoo/1 L distilled water), 0.2 L/crew-day of oral hygiene (8 g of Arm & Hammer toothpaste/0.8 L distilled water), and 0.0375 L/crew-day of shaving gel (0.8 g of Neutrogena gel/0.15 L distilled water) [22].

To evaluate the graywater MABR as the only storage tank for the graywater stream and determine the reactor’s treatment ability in case of volume fluctuation, the HC was continuously (24 h) added to the MABR, while the hygiene and laundry were pumped to the reactor as pulse events. The hygiene wastewater was added twice daily (14.5 L/event) before and after an 8 h sleep period, mimicking crew shaving, brushing teeth, washing hands, and showering before or after sleeping. Laundry was added to the reactor as a pulse (<5 min) twice weekly (15 L/event). A constant volume of 41.5 L of effluent was removed once per day after the 16 h wake period by gravity from the reactor effluent zone, except for the configuration testing the inclusion of MFM modules (Figure 2B), in which effluent was continuously removed over 24 h (Section 2.2.2).

### 2.2. Treatment System Operation

#### 2.2.1. Graywater MABR-RO System with Recycling Tank

**Figure 5:**
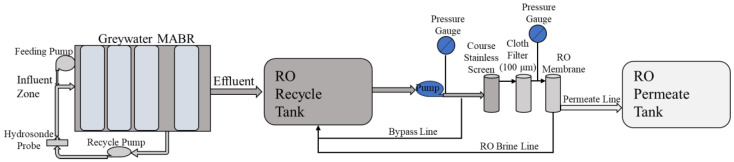
Graywater MABR and RO system diagram.

The graywater MABR effluent was pumped through the RO system at a constant RO module inlet pressure of 45 psi with a flow rate ranging from 6 to 1.8 L/h. The RO permeate was collected in a separate conical tank, while the brine from the RO module was recycled back to the feeding tank. The system was operated until 90% of the influent was collected in the RO permeate tank. The remaining 10% (brine) was transferred to the distillation system, and after each batch of MABR effluent was processed, the RO system was flushed with 10 L of permeate. This flush water was reprocessed in the next RO run. The system processed either one or two days of MABR effluent per batch. The RO module pressure, permeation rate, and recycle rate were monitored and recorded manually and periodically for each batch (Figure 2A and Figure 5).

#### 2.2.2. Graywater MABR-RO system with Membrane Filtration Module (MFM)

**Figure 6:**
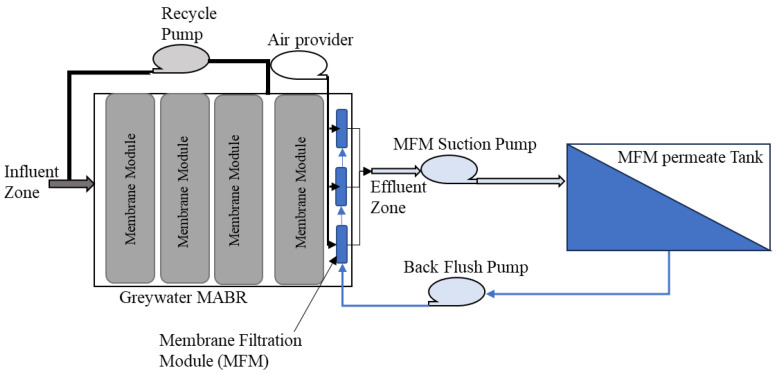
Graywater MABR-RO system flow diagram with membrane filtration module.

Figure 6 shows the ZeeWeed ultrafiltration hollow fiber membrane configuration. The ZeeWeed hollow fiber membranes form a physical barrier to solids, bacteria, and viruses. As wastewater flows through the pores and into the membrane fibers, the solids, bacteria, and viruses are blocked, letting only the treated wastewater pass through to the system. After finishing the evaluation of configuration A, the ZeeWeed membrane filtration modules (MFMs) were installed in the graywater MABR effluent zone. A peristaltic pump was connected to the MFM’s effluent side to suck the MABR effluent through a hollow fiber membrane at a desired flux (57.6 L/m^2^.day). The graywater MABR was fed in the same way as mentioned above daily, and effluent was discharged through the MFM system at a desired volume (41.5 L/day). The permeate was collected continuously in a clean tank, and 100 mL of permeate was back-flushed every hour for one minute. The MFM modules were also connected to an air pump, which was continuously operated to provide coarse bubble scouring of the outside surface of the membranes. The collected permeate (41.5 L) was processed through the RO system once per day, as described above, at a flow rate ranging from 6.6 to 2.23 L/h. Only the DOW membrane was tested for this experiment.

### 2.3. Treatment System Testing and Analysis

Samples for water quality (e.g., anions and cations) were collected from the graywater stream constitutive (e.g., laundry, humidity condensate, laundry, etc.) every day at the beginning of the experiment. After six months of operation, this process was adjusted to three times per week. The effluent samples were collected from the effluent immediately after transfer to the RO recycle tank. A portable Hach H11d Multiparameter pH/ORP meter was used to measure pH and DO in the reactor. Samples were also collected from the final permeate and brine.

All collected samples were filtered (pore size of 0.45 μm) and stored at 4 °C until further analytical process. A Dionex Ion Chromatography (IC) instrument was used to analyze anions of interest (chloride (Cl^−^), nitrite (NO_2_^−^), nitrate (NO_3_^−^), phosphate (PO_4_^−3^), and sulfate (SO_4_^−2^). A Shimadzu TOC analyzer evaluated total nitrogen (TN) and dissolved organic carbon (DOC). All DOC samples were acidified at least 24 h before analysis to remove inorganic carbon. In addition, biological oxygen demand (BOD) and chemical oxygen demand (COD) tests were performed twice a week for the influent and effluent of the reactor and RO permeates to evaluate the water quality and removal performance.

## 3. Results

We evaluated the ability of an MABR-RO system to treat a wide variety of proposed or existing space-based graywaters as part of an overall closed-loop water recycling system. The graywater MABR was used to treat combined PGH wastewater (without urine plus flush water), and the RO system was used to desalinate the reactor effluent. The graywater MABR was evaluated for OC and ON oxidation efficiency and transformation rates with respect to the waste stream and influent loading rates, and the RO system was examined for permeate quality, production rate, and consumable consumption with two different commercial membranes, as discussed in detail below.

### 3.1. Graywater Membrane-Aerated Biological Reactor (MABR) Overall Performance

**Figure 7:**
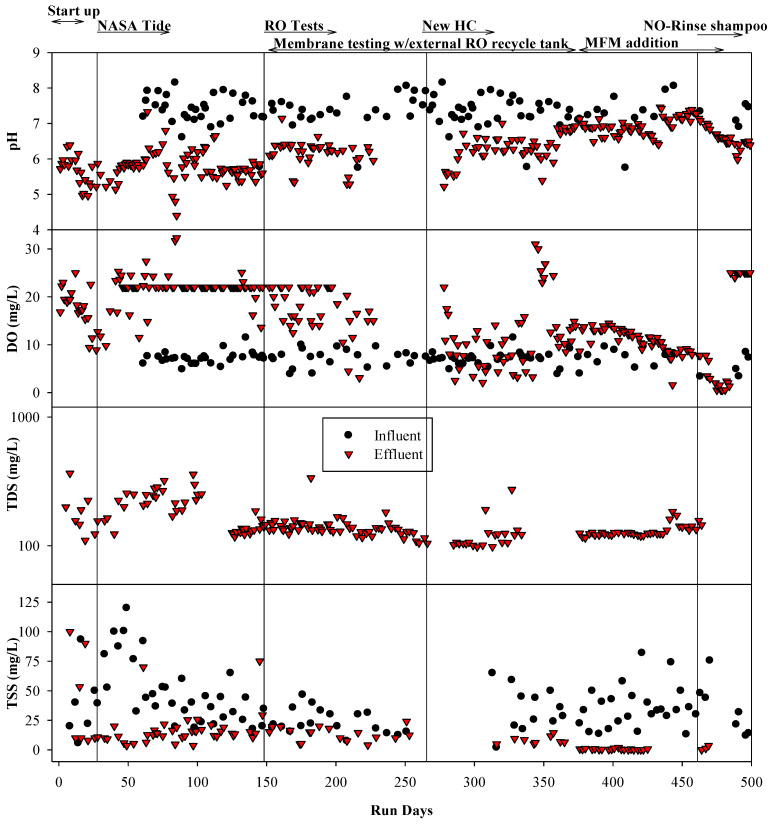
Graywater MABR influent and effluent quality during treatment of PGH graywater. NASA Tide (new formula), RO system tests, new humidity condensate (HC), and new NO-Rinse shampoo started on days 30, 148, 260, and 461 of operating the Graywater MABR.

**Figure 8:**
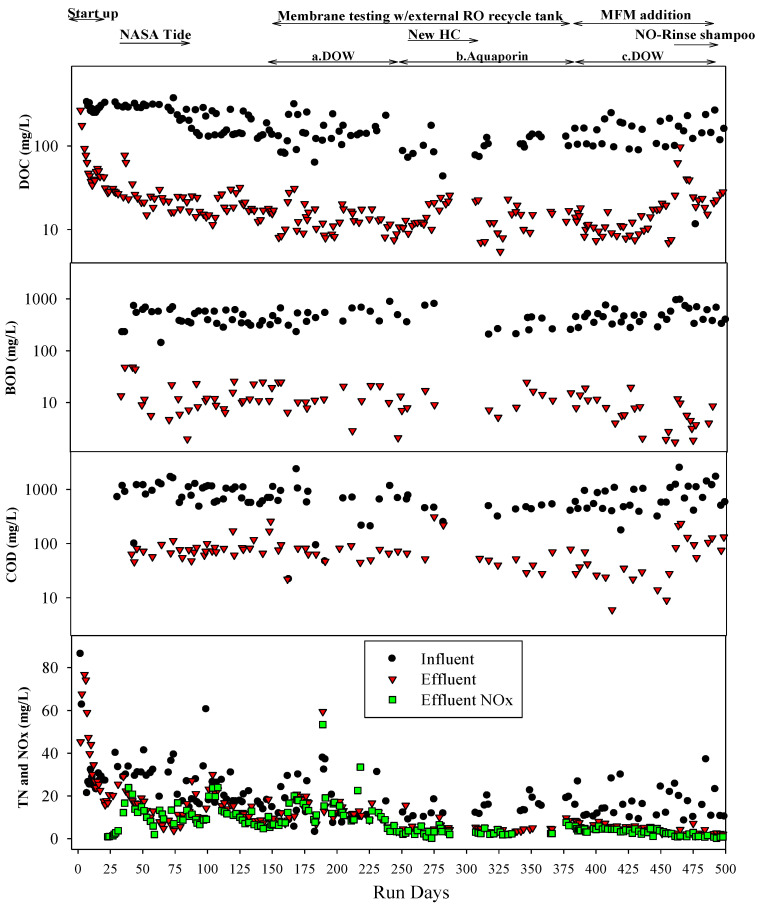
Influent and effluent water quality of an MABR treating PGH graywater.

The graywater MABR was operated for about 1.5 years. Over this time, the reactor treated ~20,750 L of combined graywater. The average pH of the reactor was ~6.5 but varied from 5 to 7.5, while the influent ranged from 7.5 to 8 (Figure 7). The DO in the reactor varied from ~3 to >25 mg/L, with higher concentrations over the first 250 days. DO was variable based on the time of day taken and pulse loading events. The influent DOC, TN, BOD, and COD concentrations ranged from 109 to 160, 15 to 18, 465 to 506, and 565 to 830 mg/L, respectively, over the period of operation. Concentration varied due to normal variations in clothes and the feeding regimen (laundry two times/week) (Figure 8). Usually, the higher concentrations of DOC, TN, BOD, and COD occurred on days when laundry waste was added. Systematic changes included updates to the HC recipe and the use of a new NO-Rinse shampoo, neither of which caused noticeable changes in influent quality. The graywater MABR average effluent DOC, BOD, and COD were largely stable over the operational period after the first 50 days and averaged 15.5, 10.5, and 54.5 mg/L, respectively, which represents a >90% reduction. Also, these results suggested that a cytotoxic synthetic anionic surfactant, the linear alkylbenzene sulfonate (LAS), widely used in surfactants, was degraded by more than 90%. TN effluent values were generally low (<10 mg/L), and almost all N was oxidized (NO_x_^−^) (Figure 8). The ammonia concentrations were relatively low (<20 mg/L) and as such, no external materials other than graywater were added. The system was operated for 500 days without any sludge or biomass removal. TSS effluent concentrations were generally low (<10.5 mg/L). Overall, the graywater MABR produced a high-quality effluent with high biological stability based on the low DOC and BOD concentrations and nearly fully oxidized nitrogen. It was able to function as the sole collection tank and could handle pulse additions even from high-volume events (laundry and shower waste streams).

### 3.2. Reverse Osmosis System

#### 3.2.1. Performance Using an External RO Recycle Tank and Impact of Commercial Membrane Type

After 130 days of operating the graywater MABR, RO system tests were started using the system configuration shown in Figure 4, in which the effluent of the MABR was discharged to a separate RO recycle tank once per day. The RO system was operated in batch mode to achieve 90% recovery in the permeate. The first test evaluated two commercial membranes (DOW (FilmTec-BW60-1812-75) and Aquaporin Inside^®^ (DWRO 1812)) for capacity and performance. Each membrane was tested for about three months. The test was stopped when the system could not process a 4 crew-day load (41.5 L/day) in less than 24 h. The RO module pressure was kept constant at 45 psi, and permeate flux was allowed to decline.

**Figure 9:**
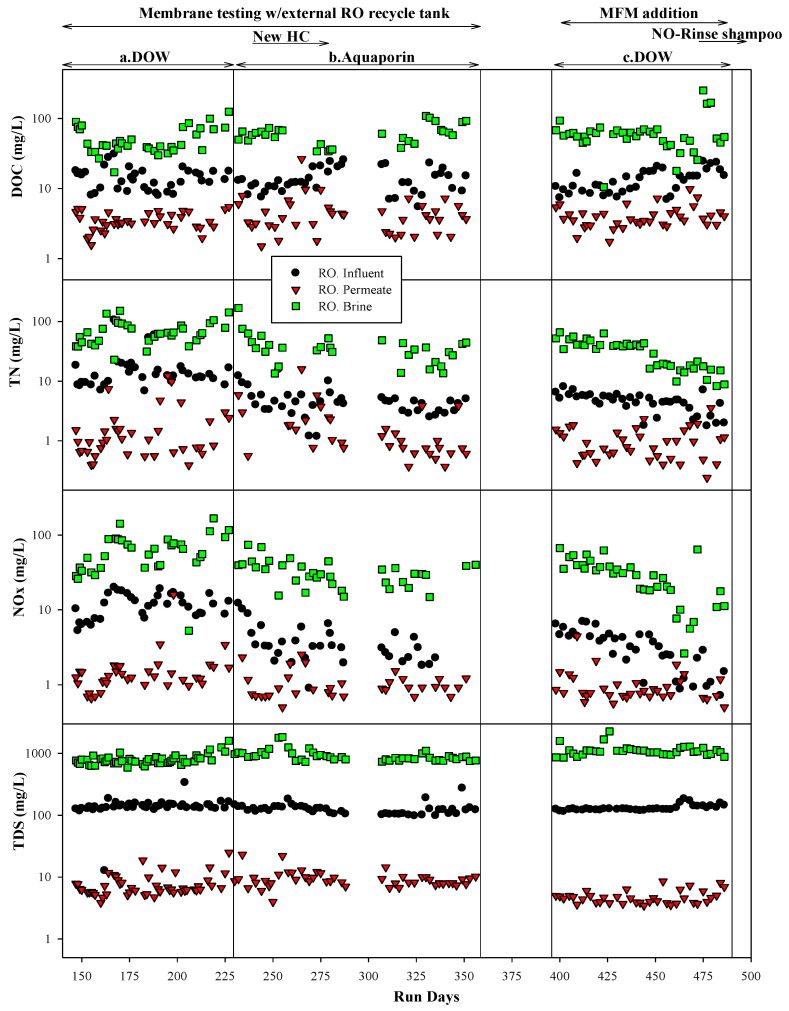
RO system water quality performance over all tests. Tests included a system with an external RO recycle tank with two different membranes ((**a**) DOW membrane and (**b**) Aquaporin membrane) and a system with an external RO recycle tank but an MFM installed in the MABR ((**c**) DOW membrane).

Graywater MABR effluent (RO influent) DOC, BOD, COD, and TDS values were similar during the testing of both membranes (Table 2). Average TN and TSS concentrations were higher (but still within one standard deviation) during the testing of the DOW membrane than during the Aquaporin. As almost all TN was present as NO_x_^−^, it is possible that the lower TN values were due to nitrate reduction as DO concentrations were lower during the Aquaporin membrane test. While DO was generally high enough in the bulk liquid to inhibit denitrification, it is possible that in the biofilms, the DO was lower. The concentrations of all parameters were fairly constant during each test (Figure 9). The concentrations of DOC, BOD, TN, NO_x_^−^, and TDS in the RO permeate were very similar over time and between membranes (Figure 9 and Table 2). There did not appear to be any change in RO permeate quality over the runtime for either membrane. For both membranes, the permeate quality was very good, with average DOC, TN, BOD, and TDS permeate concentrations of 4, 2, 5, and 8 mg/L and 5, 2, 5, and 10 mg/L for the DOW and Aquaporin, respectively (Table 2). These concentrations represent a ~98% reduction in DOC, BOD, and COD and a 95% reduction in TDS, which would significantly reduce the downstream fouling potential and the consumption of mixed beds in the WPA, major issues for the ISS.

**Figure 10:**
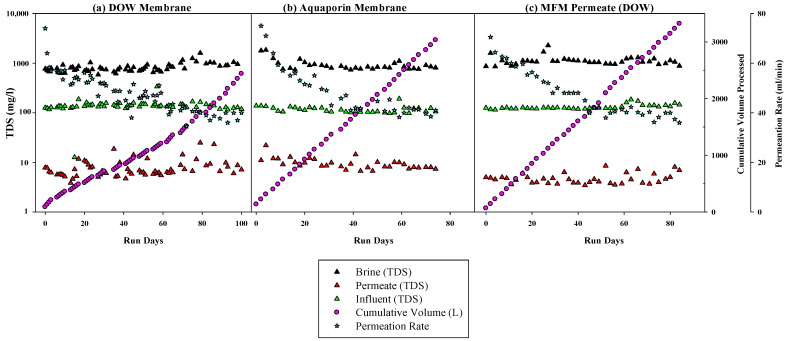
RO system performance with two different membranes: (**a**) DOW membrane, (**b**) Aquaporin membrane, and (**c**) DOW membrane during MFM setup.

The DOW membrane processed 2534 L (245 crew-days) of treated graywater, and the Aquaporin membrane processed 2771 L (268 crew-days) prior to test cessation due to the permeate production rate dropping below 37.5 L/day, or 41.5 L/day of RO influent (MABR effluent). Weekly permeate production was the same for both membranes (187.5 L/week), but the DOW membrane was operated five days per week for 11 weeks (77 days) and processed 41.5 L/run wherever this operation was switched to three times per week for the rest of the test and system production was 83 L/run (Figure 10). In general, permeate flux declined in a similar manner for both membranes, although the DOW flux (2.4 L/h) was somewhat more stable at the end of the run compared with the Aquaporin membrane and resulted in a longer operation time. It should be noted that although the test was terminated when the production rate dropped below 2.1 L/h, the flux rate (37.5 L/d) was still almost 50% of the new clean water rate, and both membranes still had significant capacity remaining. Operation with two membranes (parallel or lead-lag) could allow for even longer runs prior to replacing the membranes.

#### 3.2.2. The Membrane Filtration Module (MFM) Permeate as an RO Influent

After 230 days of the MABR treating graywater, MFMs (Section 2.2.2) were installed in the reactor effluent zone (Figure 6) to evaluate if the inclusion of MFMs would improve MABR effluent quality and increase the RO membrane life. The influent in the system was similar to that prior to the installation of the MFMs. As previously discussed, the MFM permeate (graywater MABR effluent) water quality was very similar to that prior to MFM installation, with the exception of the TSS, which dropped from 10.5 mg/L to <1 mg/L. For this test, the MABR produced effluent constantly, which was stored in one of two alternating RO recycle tanks until sufficient volume was available to process. Only the DOW membrane was tested for this experiment.

Graywater MABR-MFM effluent was processed using the RO system (DOW membrane) 5 days a week (41.5 L/day) at a permeate recovery rate of 90%. Permeate water quality was similar to or slightly better than permeate water quality for prior tests with the MFM installed in the MABR. The influent TDS was increased at the end of the test period, apparently in response to an increase in RO influent TDS caused by the new NASA NO-Rinse shampoo formula, but it was still lower than previous test points in the RO permeate (Figure 9 and Figure 10). The RO system processed a total of ~3500 L of effluent and produced 3150 L (304 crew-days) of permeate. The permeate flux decline with time was very similar to the flux decline without the MFM installed in the MABR, even though the TSS was an order of magnitude lower with the MFM. This suggests that the flux decline is likely due to membrane fouling from dissolved constituents (DOC), and the MFM may not offer substantial advantages when increasing system complexity and operation.

## 4. Discussion

To date, life support systems on the ISS or planned for upcoming moon missions have not included biological reactors for wastewater treatment despite their ubiquitous use for the treatment of terrestrial wastewaters. However, the new focus on PGHs reduces the required complexity of treatment systems compared with micro-gravity, and the likely addition of large volumes of wastewater with surfactant loads (e.g., laundry and shower) makes the current ISS wastewater treatment system inappropriate due to a number of reasons, including foaming due to surfactants, increased consumable requirements due to the use of non-regenerative systems (e.g., mixed beds), the complexity of the system, and sensitivity to failures from precipitation and/or biological fouling. The inclusion of large volumes of graywaters, which are produced in a relatively short time period, would also require the addition of storage tanks. Engineering the storage tanks to allow for biological treatment would allow for substantial reductions in contaminant loading without significant additional mass costs and allow for the inclusion of RO systems. Previous work has primarily focused on using biological reactors for the treatment of either U+F or combined U+F and graywater [15,17,18,19]. In these studies, MABRs were able to convert ~90% of the organic carbon and oxidize 50–70% of organic N into NO_x_^−^. Reaction rates were generally very low (<170 g-N/m^3^-day) due to high total ammoniacal nitrogen concentrations (600–4000 mg/L), and the need to engineer the system to operate without the need for solids processing, which would require additional complexity and system costs [19]. Separating U+F from graywater produced a high-volume and high-quality resource that was amenable to RO. To the best of our knowledge, this study was the first to evaluate a hybrid treatment system for habitation graywater in which biological processors were directly coupled to desalination systems.

Generally, this study demonstrated that an integrated graywater MABR and small-scale commercial RO system was able to treat all existing or proposed habitation graywater (HC, shower water, laundry waste, and hygiene activities). The MABR was able to be used as the only wastewater collection tank and was able to handle large-volume pulse loads (laundry and shower) while maintaining a high-quality effluent (DOC < 15 mg/L; BOD < 13 mg/L). The system stably operated for 500 days without intervention. The small-scale RO system was able to produce near-potable water (defined as NO_3_^−^ = 1.27 mg/L, NH_3_^−^ = 1 mg/L, and TOC = 3–5 mg/L) [2,4,24,25] from the MABR effluent for up to ~268 crew-days, producing between 2500 and 3000 kg of treated water.

The system has an un-optimized mass and volume (Table S2) of around 128.5 kg and 211 L, respectively, excluding the volume of pumps, filters, and accessories, which are much smaller. Consumables include O_2_ (~4 g/crew-day) and RO membranes and prefilters (1.7 g/crew-day). For a one-year mission, the total system + consumable mass is 138.5 kg, which would produce ~15,150 kg of treated water, resulting in a pay-back period of 13.4 days (3.35 days for a crew of four). Given that the MABR in this study operated for 500 days, while in previous studies, similar systems operated for up to 3 years, the total system costs would be exceedingly low. Conservatively assuming a 3-year lifespan for the MABR, the mass of the system + consumables would be 157 kg, and this system would produce ~45,440 kg of near-potable water for a total cost of 0.003 kg/kg of treated water. While it is true that the produced water would still need additional treatment to be potable, including further reductions in OC and disinfection, it could be directly useable for laundry and toilet flushing. It should also be noted that the graywater MABR reactor does not require complex control systems; is very simple, requiring only two pumps; does not use diffuse aeration, which can lead to the volatilization of off-gases; and does not produce excess solids that would need further processing. These results highlight the potential application of hybrid treatment systems for space habitats, which may also have a direct application to terrestrial applications where source-separated systems are employed.

## Figures and Tables

**Table 1:** Partial gravity habitation waste stream compositions and volumes.

Waste Stream		Daily Volume for 1 Crew Member (CM)	Daily Volume for Crew of 4 (L/day)
		(L/CM-day)	
Flushed Urine (ISS wastewater)		2.3	9.2
Hygiene	Oral hygiene (Arm & Hammer, 8 g)	0.2	0.8
	Hand wash	1	4
	Shower	6	24
	Shave (Neutrogena, 0.8 g)	0.0375	0.15 (1 CM shave/day)
Laundry (2 loads/week, 30 L-wastewater/week)		1.1	4.4
Total graywater		8.3	33.3
Humidity condensate		2	8
Total wastewater		10.35	41.3

**Table 2:** Average results for all test points for graywater MABR influent, effluent (RO influent), RO permeate, and RO brine. All data are reported as a mg/L.

Test Point		Membrane Comparison with External RO Recycle Tank				MFM Test with External RO Recycle Tank	
Membrane		a. DOW		b. Aquaporin		c. DOW	
Results		Average	STDEV	Average	STDEV	Average	STDEV
Graywater MABR Influent	DOC	160	57	110	40	160	80
	TN	18	9	15	5	15	7
	TDS	160	50	140	23	160	44
	TSS	28	12	21	17	38	17
	BOD	470	140	480	200	510	180
	COD	680	500	560	200	830	480
Graywater MABR Effluent (RO Influent)	DOC	14	5	14	5	19	18
	TN	12	4	8	5	5	1
	TDS	140	32	130	32	130	15
	TSS	17	14	11	5	1	2
	BOD	13	7	12	6	7	5
	COD	85	52	84	80	70	60
RO Permeate	DOC	4	1	5	4	4	1
	TN	2	2	2	2	1	0.5
	TDS	8	4	10	3	5	1
	TSS	5	5	4	2	0	0
	BOD	5	2	5	2	3	2
RO Brine	DOC	54	22	64	26	94	60
	TN	69	33	30	11	34	25
	TDS	830	170	920	240	1100	260
	TSS	70	200	57	110	78	120

## Data Availability

Data is available upon request.

## Supplementary Materials

The following supporting information can be downloaded at https://www.mdpi.com/article/10.3390/membranes14060127/s1: Table S1: Humidity condensate recipe.; Table S2: Graywater MABR and RO system mass and volume details.
