# Supplementary material for: Integration of Full-Size Graywater Membrane-Aerated Biological Reactor with Reverse Osmosis System for Space-Based Wastewater Treatment
Source: Membranes (Basel). 2024 May 30;14(6):127. doi: 10.3390/membranes14060127 (PMC11205946; doi:10.3390/membranes14060127)
Supplement: Supplementary file 1 [file membranes-14-00127-s001.zip › membranes-3005380-supplementary.pdf]

membranes

MDPI

Supporting Information

Integration of Full-Size Graywater Membrane-Aerated Biological Reactor with Reverse Osmosis System for Space-Based Wastewater Treatment

Ghaem Hooshyari, Arpita Bose and W. Andrew Jackson *

Table S1. Humidity condensate recipe.

Chemical name	HC Ersatz		Chemical name	HC Ersatz	
**Inorganic**	[mM]	mg/L	**Organic**	[mM]	mg/L
Zinc Acetate	121	15	Ethanol	1,449	67
Nickel Acetate	50	5.9	Acetate Ion	see inorganics	
Ammonium bicarbonate	2,509	198.4	DMSD (uses 50th percentile)	401	40
Ammonium acetate	329	14.8	Propylene glycol	357	27
Ammonium formate	91	2.9	Methanol	204	6.5
Ammonium fluoride	37	1.4	Benzyl alcohol	135	15
Monopotassium phosphate	4.3	0.59	Formate Ion	see inorganics	
Calcium bicarbonate	8.4	1.4	Ethylene glycol	73	4.5
Sodium bicarbonate	4	0.3	Acetone	45	2.6
			Caprolactam	21	2.3
			2-Propanol (Isopropanol)	17	1
			Benzoic acid	16	2
			2-Phenoxyethanol	14	2
			2-(2-Butoxyethoxy) ethanol or Diethylene glycol butyl ether	12	2
			N,N-Dimethyl acetamide	11	0.9
			Diethyl phthalate	5.4	1.2
			Trimethylsilanol	4.6	0.41
			Acetaldehyde	3.7	0.16
			Formaldehyde (assuming 100% purity)	2.7	0.08

Table S2. Greywater MABR and RO system mass and volume details.

Component	Quantity	Unit Dry Mass (Kg)	Unit External Dimensions (cm)		Unit Volume (L)
Greywater MABR	1	110	Length	80	169
			Width	45	
			Height	85	
Recirculation Pump	1	3.5	Length	16	
			Diameter	10	
Mass Flow Controller	4	0.585	Length	6	
			Width	3	
			Height	10	
RO Recycle Tank	1	8	Length	71	41.5
			Diameter	66	
RO Pump	1	3	Length	20	
			Diameter	10	
Screen Filter	1	1	Length	20	
			Diameter	6.5	
Cloth Filter	1	1.07	Length	30	
			Diameter	11.5	
RO Membrane Module	1	0.56	Length	30.5	
			Width	3.5	

Disclaimer/Publisher's Note: The statements, opinions and data contained in all publications are solely those of the individual author(s) and contributor(s) and not of MDPI and/or the editor(s). MDPI and/or the editor(s) disclaim responsibility for any injury to people or property resulting from any ideas, methods, instructions or products referred to in the content.
